# Effects of Endurance and Resistance Training on Calcitonin Gene-Related Peptide and Acetylcholine Receptor at Slow and Fast Twitch Skeletal Muscles and Sciatic Nerve in Male Wistar Rats

**DOI:** 10.1155/2012/962651

**Published:** 2012-06-18

**Authors:** Abdolhossein Parnow, Reza Gharakhanlou, Zeinab Gorginkaraji, Somayeh Rajabi, Rasoul Eslami, Mahdi Hedayati, Reza Mahdian

**Affiliations:** ^1^Exercise Physiology Department, School of Physical Education, Razi University of Kermanshah, P.O. Box 6714414874, Kermanshah, Iran; ^2^Physical Education Department, Humanity Faculty, Tarbiat Modares University, Tehran, Iran; ^3^Physical Education Faculty, Al-Zahra University, Tehran, Iran; ^4^Obesity Research Center, Research Institute for Endocrine Sciences, Shahid Beheshti University of Medical Sciences, Tehran, Iran; ^5^Department of Molecular Medicine, Biotech Research Center, Pasteur Institute of Iran, Tehran, Iran

## Abstract

The aim of this study was to investigate effects of endurance and resistance training (ET and RT) on CGRP and AChRs at slow and fast twitch muscles and sciatic nerve in rats. Twenty-five male rats were randomly assigned into three groups including sedentary (SED), endurance training (ET), and resistance training (RT). Animals of ET exercised for 12 weeks, five times/week, and 60 min/day at 30 m/min. Animals of RT were housed in metal cage with 2 m high wire-mesh tower, with water bottles set at the top. 48 h after the last session of training protocol, animals were anaesthetized. The right sciatic nerves were removed; then, Soleus (SOL) and Tibialis anterior (TA) muscles were excised and immediately snap frozen in liquid nitrogen. All frozen tissues were stored at −80°C. Results showed that, after both ET and RT, CGRP content as well as AChR content of SOL and TA muscles significantly increased. But there was no significant difference among groups at sciatic nerve' CGRP content. In conclusion, data demonstrate that ET and RT lead to changes of CGRP and AChR content of ST and FT muscles. The changes indicate to the importance of neuromuscular activity.

## 1. Introduction

Calcitonin gene-related peptide (CGRP), generated from the calcitonin gene [1–3], is distributed in the peripheral and central nervous systems of vertebrate and invertebrate species [4, 5]. CGRP's target organs are numerous, and its range of biological actions is extensive; for instance, CGRP is a very potent vasodilator [4–7], possesses positive chronotropic and inotropic effects [8], modulates neurotransmission in central [9] and peripheral [9] synapses, and modulates systemic circulation [4, 10]. 

In the peripheral nervous system, CGRP coexists with ACh in motoneurons [3], and studies have shown that CGRP_*α*_ enhances the expression of the acetylcholine receptor  (AChR)_*α*_-subunit mRNA in skeletal muscles [11], and prolongs the mean open time of AChR channels [12]. Other studies also have indicated that motoneuron CGRP_*α*_ but not CGRP_*β*_ is upregulated by axotomy or blockade of neuronal activity, suggesting that this particular peptide plays a role in motoneuron regeneration [13, 14]. These results, therefore, suggest that CGRP_*α*_ acts as an anterograde “trophic” agent that controls the synthesis and function of muscle AChRs via cAMP-mediated pathways at the neuromuscular junction (NMJ) [9, 15]. Its proposed effects at the NMJ include prolonged mean open time of AChR channels [13] and increased desensitization of AChR via a phosphorylation mechanism in the short term [16–19] and increased synthesis of AChR via a cAMP-associated pathway in the longer term [20, 21]. These observations support the idea that the peptide may also participate in the control of G4 acetylcholinesterase (G4 AChE) at the NMJ [16]. Fernandez and Hodges-Savola have indicated that motoneuron-derived CGRP_*α*_ plays a key role in the trophic control of AChE molecular forms in adult NMJs [22].

Studies have shown that motoneurons supplying fast-twitch muscles (e.g., EDL) show higher levels of CGRP staining than do motoneurons innervating muscles of slow-twitch (ST) fiber (e.g., SOL) [18–20, 23]. A similar pattern of CGRP expression is observed in the muscle, with CGRP found predominantly at the motor end plates (MEPs) of fast-twitch (FT) fibers [24]. However, it is not clear whether muscle type has contribution in CGRP release or not. 

It has been postulated for many years that an increase in neuromuscular activity has resulted in an increased motoneuronal CGRP. Homonko and Theriault showed an increased CGRP in the motor neurons of medial gastrocnemius muscles in rats 72 hours after eccentric downhill running. Their results may indicate a preferential response of FG fibers after unaccustomed exercise, resulting in synaptic reorganization [25]. In addition, it has been reported that one 30-min bout of downhill running resulted in increased numbers of CGRP+ motoneurons in hindlimb extensor but not flexor motor nuclei [26]. Jonhagen et al. reported that an increased CGRP observed after hard eccentric exercise was related to increased experience of pain [27]. Forsgren et al. have suggested that an increased neuromuscular activity in the form of regular endurance training (ET) leads to an increased CGRP in the soma and axon [28].

However, there is no evidence of RT-induced CGRP and AChRs possible changes in muscles and sciatic nerve. Thus, we have investigated the content of CGRP of slow and fast twitch muscles and the sciatic nerve, following ET and RT protocols.

## 2. Material and Methods

### 2.1. Animals

Twenty-five Wistar rats (200–250 g, 10 mo), purchased from Pasture Institute (Tehran, Iran) and maintained in the Animal House, School of Medical Sciences of Tarbiat Modares University (TMU). The animals were housed four per cage with volume of 46 L. The light-dark cycle was 12 h. Temperature was 22 ± 1.4°C, and humidity was 55.6 ± 4.0%. Animals were fed with a pellet rodent diet and had free access to water. Animals were randomly assigned into three groups including: sedentary (SED) (*n* = 8), endurance training (ET) (*n* = 8), and resistance training (RT) (*n* = 9). Ethics Committee of TMU approved the experimental protocol, and their Guidelines for Care and Use of Laboratory Animals were followed.

### 2.2. Endurance Training (ET)

ET began with familiarization of rats with the apparatus for 1 wk by placing them on the motorized-driven treadmill. In the first week, animals were exercised on treadmill at 10 m/min speed, 0% inclination, and 30 min/day. During next weeks, the load of training gradually increased up to 30–60 m/min, at a 0% inclination, 10–60 min/day, 5 days/week (Table 1). This condition corresponds to intensity about 80% of VO2_max⁡_ [29]. 

### 2.3. Resistance Training (RT)

Rats of RT were housed in metal cage with a wire-mesh tower, with two water bottles set at the top [30]. At the beginning, the bottles were set at a height of 20 cm. The set point of the drink bottles was gradually elevated to 200 cm over 1 wk. Rats were monitored for 24 h/day every 2 wk during the experimental period by using a charge-coupled device video color camera (CCD-JK-219, JMK, Japan). 

### 2.4. Tissue Preparation

48 h after the last training session, animals were anaesthetized with a mixture of Ketamine (75 mg/kg-1) and Xylazine (5 mg/kg-1) which was administered intraperitoneally. The right sciatic nerves were surgically removed and frozen in liquid nitrogen. The *soleus (SOL, as ST muscle) and tibialis *anterior *(TA, as FT muscle) *[24, 31] were excised, frozen in liquid nitrogen, and stored at −80°C. Frozen tissues (70–100 mg) were powdered in a cold mortar and pestle cooled in liquid N_2_ and dry ice then divided into two parts which were used to examine CGRP and AChR content.

### 2.5. CGRP Assay

SOL and TA muscle samples and sciatic nerve samples were washed with ice-cold PBS, homogenized 1 : 10 in 10 mM PBS, pH 7.4 at 4°C, and centrifuged (20000 rpm/45 min), and CGRP content was measured. Then, CGRP content was determined using available commercial enzyme immunoassay method (SPIbio, Massy, Cedex, France) according to kit manufacture's instruction. The assay sensitivity was 5 pg/mL, and the intra-assay coefficient of variation was 7.9%.

### 2.6. AChR Assay

Levels of AChR in the homogenized (1 : 10 in 10 Mm PBS, pH 7.4 at 4°C) and centrifuged (20000 rpm/45 min) muscle samples were determined using Rat nicotinic acetylcholine receptor ELISA kit (Wuhan USCN Sciences Co., Ltd., Wuhan, China) according to the manufacturer's instructions.

### 2.7. Statistics

Results are expressed as means ± Std. E. Variables were analyzed by unpaired *t*-test and Two-way ANOVA. *P* values <0.05 were considered significant.

## 3. Results

As presented in Tables 2 and 3, partial Eta squared is 42% and 36% in the first row, respectively, for CGRP and AChR that shows a significant effect of different mode of training on muscle CGRP and AChR contents. However, it seems that type of muscle (FT versus ST) may explain about 7% of CGRP and AChR changes (not significant) at muscles.

### 3.1. CGRP Content in SOL and TA Muscles

**Figure 1:**
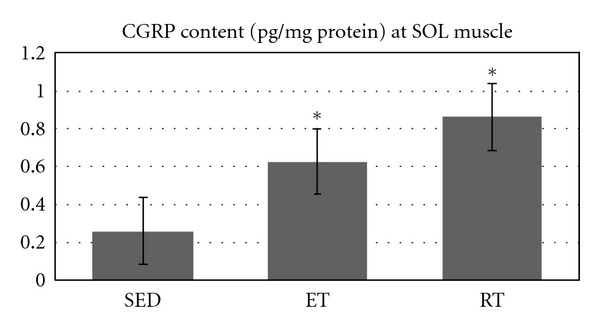
Muscle CGRP content (pg/mg protein) at slow twitch muscle in all groups. *Indicates significant (*P* ≤ 0.05) difference from sedentary group.

**Figure 2:**
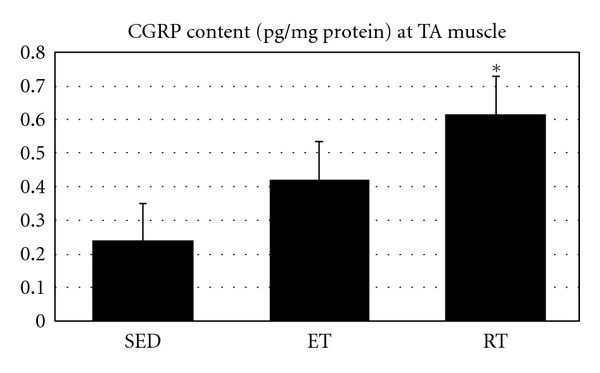
Muscle CGRP content (pg/mg protein) at Fast twitch muscle in all groups. *Indicates significant (*P* ≤ 0.05) difference from sedentary group.

**Figure 3:**
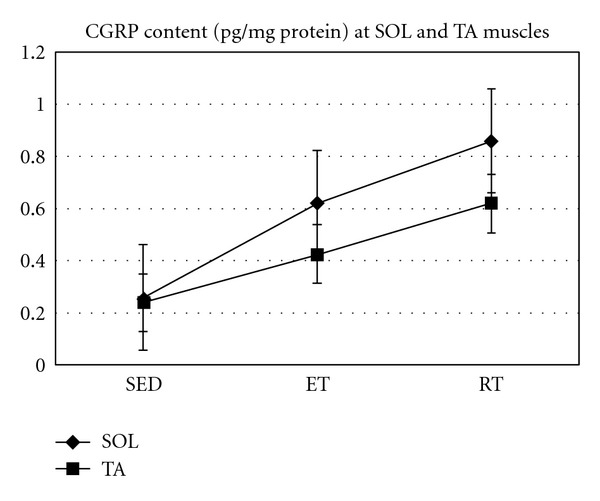
Muscle CGRP content (pg/mg protein) at slow and fast twitch muscle in all groups.

As Figures 1, 2, and 3 show, after both types of training CGRP content of SOL and TA have significantly increased (*P* = 0.001 and *P* = 0.017, resp.).

Results showed that RT leads to a significant increase in CGRP content of SOL (*P* = 0.001) as well as AT muscles (*P* = 0.013). CGRP content was increased significantly at SOL muscle by ET (*P* = 0.036), but no significant increase following ET at TA muscle was found (*P* = 0.287). However, no significant difference was observed between ET and RT at SOL muscle CGRP (*P* = 0.212), and there was also no significant difference between ET and RT regarding AT muscle CGRP content (*P* = 0.283). As data suggests there is no significant change in CGRP content at SOL and TA muscles in SED as unpaired *t*-test analysis showed (*P* = 0.984).

### 3.2. CGRP Content of Sciatic Nerve

**Figure 4:**
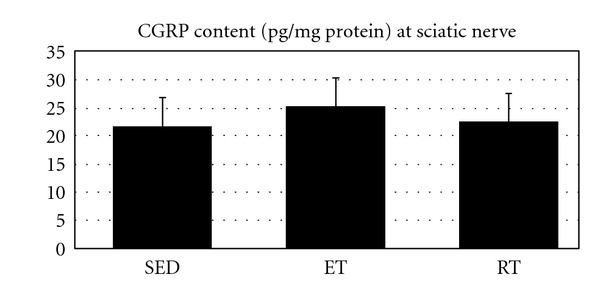
Sciatic nerve CGRP content (pg/mg protein) in all groups.

As Figure 4 shows there was no significant difference between SED, ET, and RT group's sciatic nerves (0.827). However, there is a trend at sciatic nerve CGRP content to increase following ET.

### 3.3. AChR Content in SOL and AT Muscles

**Figure 5:**
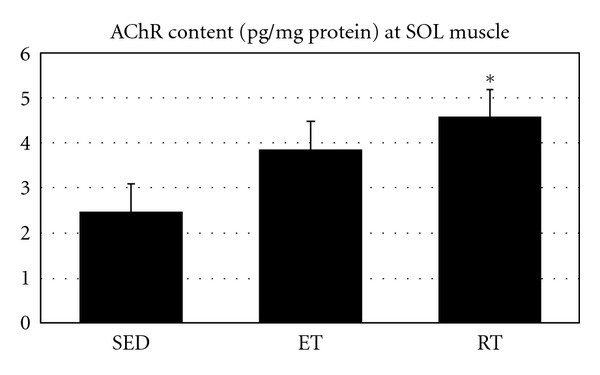
Muscle AChR content (pg/mg protein) at slow twitch muscle in all groups. *Indicates significant (*P* ≤ 0.05) difference from sedentary group.

Data analysis showed that both protocols significantly increased AChR content at both SOL and TA muscles (*P* = 0.012 and *P* = 0.001, resp.). Thereby, there was a difference, but not significant, in AChR content between SED and ET at SOL muscle (*P* = 0.116) (Figure 5). In addition, RT increased AChR content at SOL muscle (*P* = 0.010) (Figure 5).

**Figure 6:**
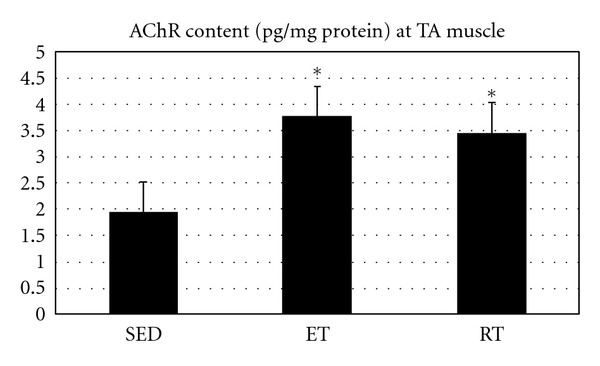
Muscle AChR content (pg/mg protein) at fast twitch muscle in all groups. *Indicates significant (*P* ≤ 0.05) difference from sedentary group.

As illustrated in Figure 6, AChR content at TA muscle was significantly different when SED was compared with ET and RT groups (*P* = 0.001 and *P* = 0.005, resp.) (Figure 6).

**Figure 7:**
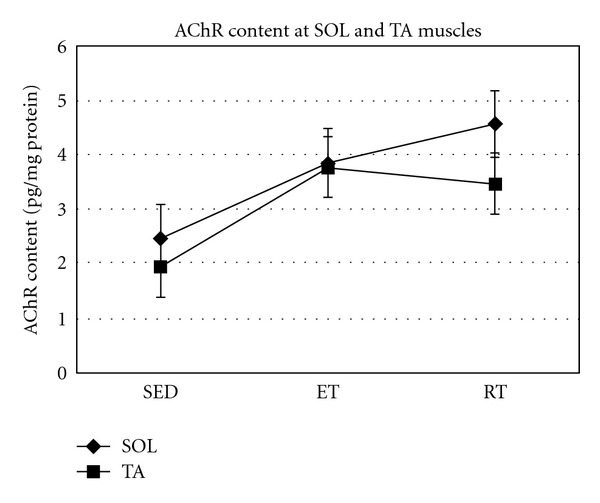
Muscle AChR content (pg/mg protein) at slow and fast twitch muscle in all groups.

As data analysis suggests, there were no significant changes between AChR content at SOL and AT muscles of SED, ET, and RT groups (Figure 7).

## 4. Discussion

The results of our study demonstrate that both ET and RT protocols have been capable of significantly altering CGRP and AChR contents of ST and FT muscles and probably CGRP of the sciatic nerve.

### 4.1. CGRP and AChR Content at ST and FT Muscles

In this study, no differences of CGRP and AChR content between ST and FT muscles of SED were observed. These results show that muscle fiber type, probably, is not the main criteria for the basal level of CGRP as well as AChR. There are, however, contradictory documents related to the fiber type-dependent differences among muscles and related motor units. For example, Osterlund et al. found that the overall distribution of CGRP immunoreactivity was similar when comparing motoneurons innervating SOL, TA, and lateral gastrocnemius [32]. Later, Forsgren et al. reported that the mean CGRP-LI staining intensity was not different when comparing motoneurons innervating the slow SOL with larger motoneurons found in the near vicinity of the SOL pool, which most likely include those innervating plantaris, TA, and gastrocnemius, all composed of primarily FT fibres/motor units [28]. In contrast, Blanco et al. found comparable CGRP mRNA levels in motoneurons innervating rat SOL, EDL, and Tensor fascia muscles [23]. Moreover, Homonko et al. have suggested that motoneurons which innervate FT muscles (e.g., LG and EDL) have higher basal level of CGRP compared with smaller motoneurons which innervate ST muscles (e.g., SOL) [25]. They also reported that fast motoneurons' CGRP level is higher than slow motoneurons [23]. These authors indicate that the main reason of these contradictory findings is related to the used protocols [25, 26]. However, findings of the present study indicate that basal level of CGRP and AChR is similar in FT and ST muscles. There are, on the other hand, some contradictory elements that provide the challenge for CGRP expression and its release such as inherence differences between ST and FT' motor units [33]. Some researchers believe that signal transport from target tissue for CGRP release is the first circle of the chain [34], and it is assumed that motoneuronal CGRP expression depends on target tissue muscle [33, 34]. The neuromuscular activity, therefore, could have a key role in CGRP releasing and expression, as it causes new functional and/or structural demands of NMJ components.

### 4.2. Endurance and Resistance Training-Induced CGRP Changes

In general, both ET and RT protocols lead to change CGRP and AChR content of ST and FT muscles. Reports have documented that both ET and RT stimulate morphological remolding of NMJ. For example, weight lifting has affected morphological profiles in NMJ architecture [4, 35]. Increased neuromuscular activity, in the form of ET, that is, running, also impacts NMJ structure [29, 30, 36]. Lu et al. showed that EDL MEPs were more extended in trained animals to compare with control animals [33]. More recently, it was shown that exercise training affected presynaptic nerve terminal branching by increasing length and complexity [35].

In accordance with other reports, in present study CGRP that is a neurotrophic and neuromodulator factor at NMJ [28, 32] was affected by neuromuscular activity in the form of ET and RT. Our results showed that CGRP content at SOL was significantly increased following ET and RT. Previously, studies have shown that tetrodotoxin-induced muscle paralysis [37], botulinum toxin-induced nerve terminal sprouting [38], and aging [19] are conditions that lead to CGRP increase. Takami et al. have proposed an upregulation of motoneuronal CGRP to occur when motor nerve terminals are either immature or unstable [38].

As described, neuromuscular activity has contribution in NMJ remodeling, and it may induce unstable NMJ. Therefore, in the present study, CGRP content probably is changed at NMJ area. Eisen et al. were the first who showed the effect of long-time ET protocol on motoneuronal CGRP in cell bodies of SOL and neighbouring motoneurons in endurance-trained rats which was significantly increased (90%) [30]. For example, Fernandez et al. reported a decrease of CGRP at muscle' nerve terminal [15]. Animals were exercised at a speed of 9 m/min and an inclination of 5°C, twice/day for 1 or 2 days. In our research, however, animals exercised at speed of 30 m/min, 5 times/day for 12 weeks. The results of our study are the first to demonstrate that CGRP content at ST and FT muscles is increased following daily RT.

Muscle CGRP content was measured after eccentric exercise by Jonhagen et al. They observed increased concentrations of CGRP after eccentric exercise together with increased experience of pain. Thus, they believed that CGRP may be involved in the regulation of DOMS after heavy exercise while simultaneously stimulating tissue regeneration [27]. More recently, Kaminski et al. showed that ET and static RT did not significantly change muscle CGRP levels after four-week training [39]. Homonko and Theriault have shown that, after downhill running in the rat, CGRP expression is elevated in MG motoneurons and at MG MEPs on FG fibers. They reported that increased CGRP levels in MG motoneurons, and subsequently at MEPs, could be due to a variety of factors such as histopathological damage to the muscle [25].

Although there is no available document investigating CGRP content change through RT, the findings of the present research might be explained by the well-known effect of RT on NMJ, and its mediators such as CGRP. Our data showed that RT has affected both ST and FT muscles. These results show that CGRP perhaps depends on the neuromuscular activity of muscle in adult animals. Although muscle CGRP was increased significantly at both ST and FT muscles by RT, the signaling mechanism is unknown.

It should be noted that contraction type probably has a key role in CGRP changes. Since eccentric contractions lead to FT fiber activity [26], and NMJ area possibly become unstable, this disruption of NMJ, therefore, could be cause of CGRP releasing. Our results showed that muscle CGRP content obviously was affected through RT compared with ET. As the drink bottles were set at a height of 200 cm, our video analysis showed that RT animals frequently did climbing activity. These animals, therefore, experienced the eccentric contractions during going down, and this fact may explain part of more changes of muscle CGRP content following RT than ET. In general, the results show that the muscle involvement and the levels of activity play a key role to compare with fiber type, as SOL response shows to be more remarkable than TA response.

### 4.3. Endurance and Resistance Training-Induced Sciatic Nerve' CGRP Changes

It is well known that spinal motoneuron cell bodies synthesize CGRP which is conveyed to nerve terminals by axonal transport [40] where stored at small vesicles and is released on nerve stimulation [41]. We studied CGRP content in sciatic nerve after ET and RT. Our data showed no change in CGRP content, and it was in disagreement with previous reports. For example, Forsgren et al. reported a significant increase (37%) in sciatic nerve CGRP of endurance-trained rats compared with SED rats [28].

There is a few research investigating CGRP content of sciatic nerve; however, it should be noted that in most of previous studies there has been a ligature on sciatic nerve by which it has been possible to show the accumulation of CGRP, as Forsgren et al. have suggested that content of CGRP sciatic nerve measured 4 h following application of a ligature [28]. We attempted to find the possible changes of sciatic nerve CGRP content without application of ligature. As the data shows that CGRP content is similar in the trained and untrained animals, one may speculate that despite of the increased level of CGRP in trained group, its axonal transportation also has been increased to facilitate the presence of this trophic factor in NMJ. It had been hypothesized that increased muscle activity causes motor axons to hypertrophy in order to meet the demands of muscle activation [42]. Eisen et al. used the soleus which is more dependent upon neurotrophic and/or neuroregulatory factors than phasic muscles. The greater tendency of axons of tonically activated muscle (soleus) to increase in diameter with hyperactivity, and decrease in diameter with hypoactivity could be explained by the greater presence of neurotrophic and neuroregulatory proteins [16]. In addition, in a study using a running protocol, radiolabeled amino acids were injected into motor neurons that supply the sciatic nerve to examine fast orthograde axonal transport and to determine its velocity. After 8 weeks of training, transport of labeled protein increased in the motor axons. In contrast, exposing untrained animals to one session of exhaustive exercise reduced total transport by 36% [3]. Thus, increase in axonal transport is an adaptive change of neurons to the requirements of chronic sustained exercise, and not an immediate response to a single training episode. These results could indicate that CGRP probably does not remain in sciatic nerve despite its axonal transport increase. It, however, needs more research to be well understood.

### 4.4. Endurance and Resistance Training-Induced AChR Changes

Our data showed all training protocols of this study significantly increased the muscle AChR content. Although possible morphological changes of NMJ were not measured in this study, findings showed that probably this aspect of NMJ remodeling is affected through the training protocols. The increased AChRs content is in coordination with other findings of the present study as increased level of muscle CGRP and, most likely, the facilitated transportation of this peptide. It is believed that exercise-induced MEP distribution has a direct relation to the number of AChRs [4]. It has been shown that chronic electrostimulation induces a drastically increased expression of specific subunits of the nAChR. This report has found that *α* and *δ* AChR subunits were increased in 10–70 day electrostimulated muscle specimens [43]. The enhancement of AChR number/MEP may have significant functional advantages. Therefore, as previous studies have reported, the functional benefits of this morphological remodeling could present a diminution of muscle fatigue during a series of high-intensity muscle contractions [4, 35].

Moreover, it is suggested that low-intensity running decreases postsynaptic development, while high-intensity running increases it [4]. Therefore, our animals were exercised at speed of 30 m/min which is high-intensity training. Thus, our data showed that ET increased AChR content at both ST and FT muscles which could be part of well-known remodeling of NMJ following this type of stimulation.

AChR content also was increased through RT of the present study as many of studies that reported RT are known to have effects at NMJ [4, 36]. Deng and Li reported that RT results in greater dispersion of AChR and vesicle clusters, respectively, within the overall NT and MEPs [4]. Such increase in AChR content of the muscle may enhance important synaptic functional capacities. Consequently, it could be speculated that these training protocols probably have improved the state of the NMJ as well as NMJ-mediated factors such as CGRP which may effect on structure and function of muscle including a delay in muscle fatigue in normal conditions [4].

CGRP has been found to colocalize with AChE and AChRs in all of the individual MEPs examined [22]. Following CGRP release, it selectively binds to high affinity; G-protein coupled receptors CGRP-Rs concentrated at the MEP [15]. CGRP controls the synthesis and function of muscle AChRs via cAMP-mediated pathways [15, 22].

In our study, data analysis showed that RT significantly increased both CGRP and AChR content at ST and FT muscles. Although we did not measure factors such as cAMP, it could be speculated that exercise-induced CGRP increase probably has been involved in AChR increase. It should be noted that investigations have shown that treatment with exogenous CGRP elevates muscle intracellular cAMP activity [44], upregulates the AChR *α*-subunit mRNA, thereby promoting the synthesis of AChR [11], enhances AChR phosphorylation [17], increases the rate of AChR desensitization [16], and prolongs the mean open-time of AChR channels [12].

In conclusion, present data demonstrate that both ET and RT lead to changes of CGRP and AChR content of ST and FT muscles. The changes were independent of muscle fiber type that indicates to the importance of neuromuscular activity in the form of ET and RT affecting the fundamental components of neuromuscular adaptations.

## Figures and Tables

**Table 1:** Endurance training protocol.

Weeks of training	1	2	3	4	5	6	7	8	9	10	11	12
Training duration (min/day)	30	40	45	50	55	60	60	60	60	60	60	60
Treadmill speed (m/min)	10	10	12	16	20	25	30	30	30	30	30	30

**Table 2:** Two-way analysis of variance for interaction effects (CGRP) (group versus muscle type).

Source	Type III sum of squares	df	Mean square	*F*	Sig.	Partial eta squared	Noncent. parameter	Observed power
Group	1.757	2	.879	13.901	.000	.423	27.802	.997
Muscle	0.177	1	.177	2.800	.102	.069	2.800	.371
Group-muscle	0.083	2	.041	.654	.526	.033	1.309	.152

**Table 3:** Two-way analysis of variance for interaction effects (AChR) (group versus muscle type).

Source	Type III sum of squares	df	Mean square	*F*	Sig.	Partial eta squared	Noncent. parameter	Observed power
Group	32.190	2	16.095	12.608	.000	.364	25.215	.995
Muscle	4.017	1	4.017	3.147	.083	.067	3.147	.411
Group-muscle	2.250	2	1.125	.881	.421	.039	1.763	.192
